# Home-based chlamydia testing of young people attending a music festival - who will pee and post?

**DOI:** 10.1186/1471-2458-10-376

**Published:** 2010-06-28

**Authors:** Rachel Sacks-Davis, Judy Gold, Campbell K Aitken, Margaret E Hellard

**Affiliations:** 1 Burnet Institute, Melbourne, VIC, Australia; 2 Department of Epidemiology and Preventative Medicine, Monash University, Melbourne, VIC, Australia

## Abstract

**Background:**

Chlamydia is most common among young people, but only a small proportion of Australian young people are tested annually. Home-based chlamydia testing has been piloted in several countries to increase testing rates, but uptake has been low. We aimed to identify predictors of uptake of home-based chlamydia testing to inform future testing programs.

**Methods:**

We offered home-based chlamydia testing kits to participants in a sexual behaviour cross-sectional survey conducted at a music festival in Melbourne, Australia. Those who consented received a testing kit and were asked to return their urine or vaginal swab sample via post.

**Results:**

Nine hundred and two sexually active music festival attendees aged 16-29 completed the survey; 313 (35%) opted to receive chlamydia testing kits, and 67 of 313 (21%) returned a specimen for testing. One participant was infected with chlamydia (1% prevalence). Independent predictors of consenting to receive a testing kit included older age, knowing that chlamydia can make women infertile, reporting more than three lifetime sexual partners and inconsistent condom use. Independent predictors of returning a sample to the laboratory included knowing that chlamydia can be asymptomatic, not having had an STI test in the past six months and not living with parents.

**Conclusions:**

A low proportion of participants returned their chlamydia test, suggesting that this model is not ideal for reaching young people. Home-based chlamydia testing is most attractive to those who report engaging in sexual risk behaviours and are aware of the often asymptomatic nature and potential sequelae of chlamydia infection.

## Background

Chlamydia (*Chlamydia trachomatis*) is the most prevalent bacterial sexually transmitted infection (STI) in the western world[1] and the most common notifiable infectious disease in Australia[2]. Rates of chlamydia infection are increasing worldwide, and Australian notification rates quadrupled from 1999 to 2008[2]. Infection is concentrated in youth, with approximately 80% of notifications being among those aged 15 to 29 years[2]. Chlamydia infection can cause significant morbidity, particularly for women: up to two-thirds of cases of tubal infertility and one-third of cases of ectopic pregnancy may be attributable to chlamydia infection[3]. Over 80% of infections are asymptomatic,[3] making screening necessary to detect and treat cases.

Despite being the population group at highest risk of STIs, young Australians know little about STIs other than HIV[4]. Only 12% of women and 5% of men aged 16-29 years in Australia who attended a doctor in 2007-2008 had a chlamydia test[5] - far lower than the testing rate of 40% amongst those aged less than 25 years estimated to be required for a rapid reduction in chlamydia prevalence in Australia[6].

Flexibility and ease of testing have been identified as potential factors in promoting chlamydia testing[7]. Home-based chlamydia testing has been trialled in the US, Europe, Australia, and elsewhere to increase screening rates and make testing more accessible[8-13]. Vaginal and penile swabs, and urine samples, have been collected at home and returned to laboratories by post for testing. These methods are acceptable to young people,[13-17] and participants in a study in the US who were tested both at a clinic and at home preferred home-testing[17].

Nonetheless, uptake of home-based testing is generally low[9]. Response rates for home-testing kits distributed by mail in the Netherlands, Sweden, the UK, South Africa, and Brazil ranged from 24-80%[11,12,18-20]. Previous researchers distributed home-testing kits at pharmacies, gyms and other community settings, with return rates of 3-38%[13,15,21,22]. In studies in which kits were advertised and could be ordered, 30-68% of people who ordered a kit returned a specimen[15,23].

Few studies have collected information on correlates of home-based chlamydia testing uptake. In two studies, common reasons for declining testing included being in a steady relationship,[18] consistent condom use,[18] not being sexually active,[18,19] and lack of interest in testing[19]. In one study, univariate analysis found that non-respondents were less likely to report symptoms consistent with STIs or to have ever had sex, and sexually active female non-respondents were more likely to have used a condom at last sexual contact (no multivariate analysis was conducted)[19].

Given that uptake is low, knowledge of predictors of uptake of testing among sexually active young people is required to appropriately target home-based chlamydia testing to ensure program efficiency and maximise program participation. From a pragmatic perspective, the extent to which the target group for outreach chlamydia testing (young people at risk of acquiring chlamydia who are under-utilising related services) is willing to take up home-based testing is of particular importance for targeting programs efficiently. In order to maximise program utilisation, measurement of the extent to which modifiable factors (such as knowledge of chlamydia) predict uptake is also important.

This analysis aimed to identify determinants of ordering and returning chlamydia testing kits in a community setting in order to inform future testing programs. Since 2005, we have conducted cross-sectional surveys of sexual risk behaviour by people aged 16-29 attending a large annual music festival in Melbourne, Australia[24]. In 2009, we invited all participants to receive a chlamydia testing kit posted to their homes.

## Methods

### Design

A cross-sectional survey was conducted at the Big Day Out music festival in Melbourne, Australia in January 2009. Survey participants were invited to consent to receive a chlamydia testing kit by post.

### Setting and recruitment

The Big Day Out is a music festival held annually in Australasia featuring a diverse selection of music [25]. The Melbourne Big Day Out draws 40-50,000 fans, mostly young people. Our Big Day Out recruitment site consisted of a market stall positioned in the shade with chairs for participants. Participants either approached the stall or were approached by recruitment staff and asked to complete a brief questionnaire about 'sex, drugs and rock'n'roll'. Approximately 20 trained researchers recruited participants. Festival attendees were eligible for participation if they were aged between 16 and 29 years, were not intoxicated, and had sufficient English language skills to complete a self-administered questionnaire.

### Materials

Participants completed a consent form and questionnaire in approximately 15 minutes. Participants could opt to receive a home-testing kit for chlamydia by providing an additional signature and contact details for follow-up on the consent form. Consent forms and questionnaires were stored separately to protect privacy. Participants were given showbags containing answers to questions about STIs that appeared in the questionnaire, sexual health information and condoms. To encourage participation in the survey, participants were offered cold drinks and lollipops and entered in a draw to win an MP3 player and CD vouchers.

A week after recruitment, we posted chlamydia testing kits to consenting participants. The kit consisted of a sterile urine jar, a sterile flocked swab (MicroRheologies, Brescia, Italy), information about chlamydia (including testing and treatment), instructions for taking the test, and a reply-paid padded envelope for returning the specimen to the laboratory for testing. Reply-paid envelopes were labelled category C according to guidelines for posting biological specimens[26]. Two weeks after sending out the testing kits, participants who had not returned a sample to the laboratory were sent a reminder by SMS.

Negative chlamydia test results were communicated to participants by telephone, SMS, email or post (depending on preference). A trained nurse delivered positive chlamydia test results by telephone, and treatment was posted free of charge.

### Chlamydia testing

Female participants could choose to provide a self-collected vaginal swab sample or a first-void urine sample. Male participants were asked to provide a first-void urine sample. Because liquids can no longer be mailed through Australia Post, the 'self-collected urine dip swab method' was used for all urine specimens[27]. Participants were asked to hold a sterile flocked swab in a first-void urine sample for ten seconds, then package the swab and dispose of the urine. Participants providing a vaginal swab were asked to insert their swab to approximately half the length of a finger and rotate it gently. Each swab was packaged in a tube labelled with the date of collection, the participant's study ID number, date of birth, and the type of specimen (vaginal or urine dip swab), and returned to the laboratory using the reply-paid envelope provided. We informed female participants that self-collected vaginal swab tests were more sensitive than urine tests[28]. Methods for testing urine dip swab specimens[27] have been described elsewhere. Vaginal swabs were analyzed using polymerase chain reaction.

### Analysis

Our analysis included all participants who reported that they had ever had sex. Data were entered into a Microsoft Access database and statistical analysis was conducted in Stata 10. We defined inconsistent condom use as not always using condoms with new and/or casual partners, and/or regular partners if multiple regular partners were reported within the last year. A new partner was defined as someone with whom the participant had first had sex less than three months ago.

Hazardous drinking was defined as drinking more than six alcoholic drinks in a session at least weekly in the past year[29]. Australian postcodes of residence were classified according to their proximity to major cities using the Australian Standard Geographical Classification - Remoteness Areas system[30,31].

In the analysis, we considered demographic factors, known risk factors for acquiring chlamydial infection, knowledge about chlamydia and STIs, and factors relating to health service utilisation to be potential predictors of opting to receive a test and returning a completed test.

Multivariate logistic regression models were used to investigate predictors of consenting to receive a test-kit and returning a sample to the laboratory for testing. Consenting to receive a test kit was considered to be a marker of in-principle interest in home-based testing, We used univariate logistic regression to identify candidate predictors for inclusion in the multivariate models. The final model for consenting to receive a test-kit was derived through a process of backwards elimination in which non-significant variables were sequentially removed until only wholly significant predictors remained, ensuring that no confounding factor was present. Due to the smaller number of participants who returned a sample for testing, we derived the final model for returning a sample through a process of forwards elimination in which significant predictors were sequentially added to the model. Goodness of fit for both models was assessed using the Hosmer & Lemeshow test.

### Ethics

This study received ethical approval from the Alfred Hospital Human Ethics Committee in December 2008.

## Results

### Participation

Overall, 1,162 people completed the survey, 240 of whom were excluded from analysis because they reported never having had sex, and 20 because they did not disclose whether they had had sex. Of the remaining 902 participants, more than half (n = 523, 58%) were female, and the median age was 20 years (range: 16-29). Most participants (n = 549, 61%) resided in or close to major cities and were born in Australia (n = 795, 88%). Socio-demographic and behavioural characteristics of the study participants are presented in Table 1.

**Table 1:** Socio-demographic and behavioural characteristics of study participants

Characteristic	n (%)
Total number of participants	902
*Socio-demographics*	
Median age (range)	20 (16-29)
Female	523 (58.0)
Residing in or close to a major city^a^	549 (60.9)
Born in Australia	795 (88.1)
Highest level of education is high school or below	454 (50.3)
Living with their partner	125 (13.9)
Living with their parent(s)	465 (51.6)
*Reported sexual behaviours*	
Median age (range) of first sex	16 (11-25)
Median (range) number of lifetime sexual partners	4 (1-297)
Reported multiple sexual partners in the past year	426 (47.2)
Reported new sexual partner in the past three months^b^	322 (35.7)
Reported at least one same-sex partner in the past year	96 (10.6)
Reported inconsistent condom use^c^	338 (37.5)
*Reported drug and alcohol related behaviours*	
Reported hazardous drinking in the past year^d^	325 (36.0)
Reported illicit drug use in the past month	296 (32.8)
*Sexual health service utilisation*	
Reported seeing a doctor in the past six months	510 (56.5)
Reported speaking to a doctor about sexual health in the past six months	276 (30.6)
Reported having ever had an STI test (other than a pap smear)	328 (36.4)
Reported having had an STI test (other than a pap smear) in the past six months	152 (16.9)
*Level of STI-related knowledge*	
Knew that a pap smear cannot diagnose all of the main STIs	252 (27.9)
Knew that chlamydia can last for years if left untreated	654 (72.5)
Knew that chlamydia can be asymptomatic	706 (78.3)
Knew that chlamydia can be diagnosed using a simple urine test	638 (70.7)
Knew that bacterial STIs can be easily treated with antibiotics	487 (54.0)
Knew that chlamydia can make women infertile	545 (60.4)
Answered more than three of six STI-related knowledge questions correctly*	540 (59.9)

a. This classification was derived from postcode of residence using the Australian Standard Geographical Classification Remoteness Areas. All participants who provided an Australian postcode of residence but did not reside in or close to major cities, resided in inner regional areas.

b. A *new partner *was defined as someone with whom the participant had first had sex less than three months ago.

c. *Inconsistent condom use *was defined as not always using condoms with new and/or casual partners, and/or regular partners if multiple regular partners were reported within the last year.

d. *Hazardous drinking *was defined as drinking more than six alcoholic drinks in a session at least weekly.

**Figure 1:**
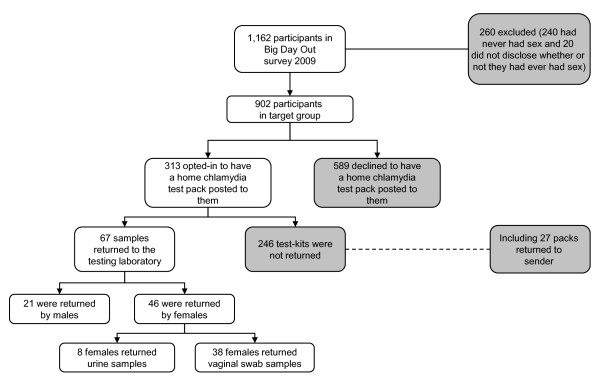
**Flow chart of recruitment and chlamydia testing participation**.

We offered all participants the opportunity to be posted a home chlamydia testing kit, and 313 (35%; 32% of male and 37% of female participants) consented. Of those, 27 kits were returned to sender indicating that the address provided was incorrect. In total, 67 (21%) tests were returned to the laboratory. Of those who completed their test, the median time between posting the test pack to the participant and the sample being received by the laboratory was 12 days (range: 3-47). Most tests returned were completed by female participants (n = 46, 69%). Among the female participants, 8 (17%) chose to return a urine sample, and 38 (83%) returned a vaginal swab sample (Figure 1). We detected chlamydia infection in one male participant (4.8% prevalence among males) and no females.

### Chlamydia testing uptake

In univariate analysis, older age, not living with parents, having had more than three lifetime sexual partners, knowing that chlamydia can make women infertile if left untreated, having more than one sexual partner in the past year, inconsistent condom use, and having used illicit drugs in the past month were predictors of ordering a home chlamydia testing kit. In multivariate analysis, older age, not living with a sexual partner, knowing that chlamydia can make women infertile, and having had more than three lifetime sexual partners were independent predictors of ordering a home testing kit. Inconsistent condom use and not reporting hazardous drinking were also marginally predictive of ordering a chlamydia testing kit (Table 2).

**Table 2:** Unadjusted and adjusted odds ratios (OR) associated with opting-in to receive a home-based chlamydia test pack^a^

		**Number (n = 902)**^**b**^	Percent ordered test	OR (95% CI)	p-value	**Adjusted OR (95% CI)**^**c**^	p-value
*Age group*	16-19	430	29.8	1.0		1.0	
	20-29	460	39.3	**1.5 (1.2-2.0)**	**< 0.01**	**1.5 (1.1-2.1)**	**0.02**
*Gender*	Male	370	31.6	1.0			
	Female	523	37.1	1.2 (0.9-1.6)	0.16		
*Living with partner*	No	748	35.7	1.0		1.0	
	Yes	125	30.4	0.8 (0.5-1.2)	0.23	**0.5 (0.3-0.9)**	**0.01**
*Living with parents*	No	408	38.7	1.0			
	Yes	465	31.6	**0.7 (0.6-1.0)**	**0.03**		
*Talking about sexual health with a doctor in the past 6 months*	Did not go to doctor	380	32.9	1.0			
	Saw doctor but didn't talk about sex	234	32.9	1.0 (0.7-1.4)	0.98		
	Talked to doctor about sex	276	38.8	1.3 (0.9-1.8)	0.11		
*Had an STI test in past 6 months*	No	743	33.9	1.0			
	Yes	152	39.5	1.3 (0.9-1.8)	0.19		
*Know that chlamydia can be asymptomatic*	No	186	33.3	1.0			
	Yes	706	35.1	1.1 (0.8-1.6)	0.51		
*Know that chlamydia can make women infertile*	No	346	29.8	1.0		1.0	
	Yes	545	37.6	**1.4 (1.1-1.9)**	**0.01**	**1.5 (1.1-2.1)**	**0.01**
*Number of lifetime sexual partners*	1-3	395	28.6	1.0		1.0	
	> 3	454	39.6	**1.6 (1.2-2.2)**	**< 0.01**	**1.7 (1.2-2.4)**	**< 0.01**
*Multiple sexual partners in the past year*	No	467	31.0	1.0			
	Yes	426	39.4	**1.4 (1.1-1.9)**	**0.01**		
*Inconsistent condom use*^d^	No	498	30.1	1.0		1.0	
	Yes	338	40.8	**1.6 (1.2-2.1)**	**< 0.01**	**1.4 (1.0-2.0)**	**0.04**
*Hazardous drinking*^e^	No	555	35.3	1.0		1.0	
	Yes	325	32.9	1.0 (0.7-1.3)	0.76	**0.7 (0.5-1.0)**	**0.03**
*Used illicit drugs in past month*	No	590	31.5	1.0			
	Yes	296	41.2	**1.6 (1.2-2.1)**	**< 0.01**		

a. The following variables were also tested as potential predictors but were not found to be significant at either the univariate or multivariate level: place of residence (urban or regional), ever having had an STI test, know that a pap smear cannot diagnose all of the main STIs, know that chlamydia can be diagnosed using a simple urine test, and having a new partner in the past three months.

b. Grouped data do not always add up to the total number of participants (902) who opted to receive a chlamydia testing kit due to non-responses.

c. Hosmer-Lemeshow: p = 0.60

d. *Inconsistent condom use *was defined as not always using condoms with new and/or casual partners, and/or regular partners if multiple regular partners were reported within the last year.

e. *Hazardous drinking *was defined as drinking more than six alcoholic drinks in a session at least weekly.

Among those who ordered a testing kit, independent predictors of completing and returning the test to the laboratory in multivariate analysis included seeing a doctor without discussing sexual health in the past six months, not having had an STI test in the past six months, knowing that chlamydia can be asymptomatic, and not living with parents (Table 3).

**Table 3:** Unadjusted and adjusted odds ratios (OR) associated with returning a chlamydia test pack^a^

		**Number ordered test**^**b**^	Percent returned test	OR (95% CI)	p-value	**Adjusted OR (95% CI)**^**c**^	p-value
*Age group*	16-19	128	18.0	1.0			
	20-29	131	23.8	1.4 (0.8-2.5)	0.22		
*Gender*	Male	117	17.9	1.0			
	Female	194	23.7	1.4 (0.8-2.5)	0.23		
*Living with partner*	No	267	21.3	1.0			
	Yes	38	23.7	1.1 (0.5-2.6)	0.74		
*Living with parents*	No	158	25.9	1.0		1.0	
	Yes	147	17.0	0.6 (0.3-1.0)	0.06	**0.5 (0.3-0.9)**	**0.03**
*Talking about sexual health with a doctor in the past 6 months*	Did not go to doctor	125	16.8	1.0		1.0	
	Saw doctor but didn't talk about sex	77	32.5	**2.4 (1.2-4.6)**	**0.01**	**2.4 (1.2-5.0)**	**0.01**
	Talked to doctor about sex	107	19.6	1.2 (0.6-2.4)	0.56	1.5 (0.7-3.1)	0.29
*Had an STI test in past 6 months*	No	252	23.8	1.0		1.0	
	Yes	60	11.7	**0.4 (0.2-1.0)**	**0.04**	**0.4 (0.1-0.9)**	**0.03**
*Know that chlamydia can be asymptomatic*	No	62	8.1	1.0		1.0	
	Yes	248	25.0	**3.8 (1.5-9.9)**	**0.01**	**3.4 (1.3-9.1)**	**0.01**
*Know that chlamydia can make women infertile*	No	103	20.3	1.0			
	Yes	205	22.0	1.1 (0.6-2.0)	0.75		
*Number of lifetime sexual partners*	1-3	113	23.0	1.0			
	>3	180	22.2	1.0 (0.5-1.7)	0.88		
*Multiple sexual partners in the past year*	No	145	22.1	1.0			
	Yes	168	20.8	0.9 (0.5-1.6)	0.79		
Inconsistent *condom use*^d^	No	150	20.7	1.0			
	Yes	138	23.2	1.2 (0.7-2.0)	0.61		
*Hazardous drinking*^e^	No	196	24.0	1.0			
	Yes	107	17.8	0.7 (0.4-1.2)	0.21		
*Used illicit drugs in past month*	No	186	20.4	1.0			
	Yes	122	23.0	1.2 (0.7-2.0)	0.60		

a. The following variables were also tested as potential predictors but were not found to be significant at either the univariate or multivariate level: place of residence (urban or regional), ever having had an STI test, know that a pap smear cannot diagnose all of the main STIs, know that chlamydia can be diagnosed using a simple urine test, and having a new partner in the past three months.

b. Grouped data do not always add up to the total number of participants (902) who opted to receive a chlamydia testing kit due to non-responses.

c. Hosmer-Lemeshow: p = 0.74

d. *Inconsistent condom use *was defined as not always using condoms with new and/or casual partners, and/or regular partners if multiple regular partners were reported within the last year.

e. *Hazardous drinking *was defined as drinking more than six alcoholic drinks in a session at least weekly

## Discussion

Our study is the first to collect detailed socio-demographic and behavioural data on all potential participants in a community-based chlamydia home-based testing pilot. We found that those at greater risk of chlamydia infection and those who were aware that chlamydia could lead to infertility were more likely to order a test. Among participants who ordered tests, those who knew chlamydia could be asymptomatic were most likely to return the test, while those who lived with their parents or had recently been tested were least likely to return the test. These findings have important implications for any future roll-out of home-based chlamydia testing.

The efficacy of home-based chlamydia testing programs is questionable, as several previous pilot programs reported low uptake[8-13]. In this study, one third of sexually active participants expressed interest in home-based testing by consenting to receive a kit, but only one fifth of those participants who ordered packs returned samples for testing. Given the low rate of chlamydia testing in primary care settings,[5] identifying alternate settings that are more attractive to the young people who are most at risk of acquiring chlamydia is likely to be an important strategy for increasing overall testing rates. Previous researchers, by offering financial incentives for chlamydia testing in a study of testing promotion at university sexual health clinics, increased uptake from 22% to 45%[32]. The low rate of participation in home-based chlamydia testing in our study suggests that financial incentives, onsite testing[33] or alternate settings might be required to make participation more attractive.

Knowledge about chlamydia has been identified as a determinant of interest in clinic-based chlamydia testing[7,34]. Our study suggests that knowledge about STIs is an important factor in decisions to order and use home-based tests; it might, therefore, be worthwhile trialling targeted educational interventions to improve uptake of home-based chlamydia testing. Being aware that chlamydia can lead to infertility was a predictor for ordering a testing kit, consistent with previous findings that knowledge of long-term sequelae of STIs is associated with related health-seeking behaviour[35,36]. Among those who ordered a test, being aware that chlamydia can be asymptomatic was predictive of using the test. This is consistent with the health belief model of behaviour change, which posits that perceiving oneself to be susceptible to the disease and belief that it can have serious consequences are determinants of health-seeking behaviour[37,38].

Our data also show that participants who ordered testing kits were at higher risk of infection because they reported more lifetime partners and were more likely to report inconsistent condom use. Those who returned swabs for testing were less likely to have had a recent STI test. These results are consistent with previous reports that young people are more likely to express interest in chlamydia screening if they have casual partners or recent sex without a condom[7,34,39]. Home-based testing, despite overall low uptake rates, may therefore be effective for reaching people at higher risk who may not be tested otherwise.

Confidentiality was previously identified as a potential detractor from willingness to participate in clinic-based chlamydia screening[7]. In our study, participants who reported living with their parents were less likely to use their testing kit. Those who lived with their parents also tended to be less likely to order a chlamydia testing kit, although this did not reach significance in multivariate analysis. This suggests that privacy might also be a barrier to home-based testing[40].

In our study, living with a sexual partner was also associated with decreased likelihood of ordering a chlamydia testing kit; privacy concerns may have prevented these participants from wanting to receive chlamydia testing kits in the post. Nevertheless, living with a sexual partner was associated with multiple factors that are protective against chlamydial infection: older age, decreased likelihood of having had multiple sexual partners in the past year, and decreased likelihood of having had new partners in the past three months (data not shown)[41]. The relatively low risk profile of those participants who reported living with their partner probably contributed to their being less likely to order a chlamydia testing kit.

Australian clinic-based chlamydia testing rates are higher among 20-29 year olds than those aged 16-19 years, and higher among women than men[5]. Thus it was unsurprising that in our study, older participants were slightly more likely to order a test than younger participants, independent of their reported number of lifetime sexual partners. In contrast, the finding that gender was neither a determinant of consenting to receive a test kit nor of returning a test kit suggests that home-based testing is an effective way to reach men who are less likely to be tested through opportunistic clinic-based screening.

Low rates of counselling about sexual health and chlamydia testing have been reported in primary-care settings in Australia and elsewhere[42-46]. In our study, almost half of sexually active participants who reported visiting a doctor in the past six months had discussed sexual health with their doctor. Of participants who ordered a test, those who had visited a doctor without discussing sexual health were more likely to return a sample to the laboratory than those who hadn't seen a doctor; this may have been because those who utilised general health services were more likely to use their chlamydia testing kit. However, we did not observe this effect among those who did talk to their doctor about sex, perhaps because these participants had either been tested or were confident after speaking to their doctor that they did not require chlamydia testing.

Those who reported having had an STI test recently were just as likely to order a testing kit but less likely to use the kit within the study period. This suggests that ordering the test was an indication of general interest in home-based chlamydia testing rather than immediate interest in being tested.

Alcohol use is often associated with increased risk of STIs in global population studies but no clear casual relationship has been established[47,48]. In this study, although the direct indicators of risky sexual behaviour were associated with being more likely to order a testing kit, hazardous drinking was associated with being less likely to order a kit.

In our study women seemed to prefer to provide self-collected vaginal swabs over urine samples, with the majority of women who provided samples opting to provide a vaginal swab. This is different to previous research [17] and may warrant future investigation if postal samples continue to be used.

Our study has some limitations. Participants were recruited using convenience sampling, and although this was advantageous in the sense that it allowed us to trial our testing strategy in a setting that could realistically be used to offer chlamydia testing in the future, it meant that our sample may not have been representative of all sexually-active young Australians. Young people attending the Big Day Out music festival may be of higher than average socio-economic status given the cost of the ticket (AU$140+booking fee). Nevertheless, we measured levels of knowledge about chlamydia similar to those observed in a national survey of Australian secondary school students in 2008[4]. Similarly, although direct comparison with health utilisation statistics was not possible, the sample appeared to access healthcare at similar rates to their peers[5].

Results from our previous Big Day Out surveys suggest that participants engage in more sex-, alcohol- and drug-related risk behaviours than other young Australians[49,50]. Given that young people who engage in sexual risk behaviour are more likely to express interest in home-based chlamydia testing, our study may over-estimate the proportion of young people interested in testing.

A low proportion of participants returned their chlamydia test, suggesting that this model is not ideal for reaching a large proportion of young people. Moreover, the small number of participants who ultimately used their home-based testing kit limited our capacity to identify predictors of returning the kit. This may have been because the urine-testing methodology used in this study was more complicated than a simple urine test[7]. Multiple potential predictors of opting to receive a chlamydia testing kit and returning completed kits were analysed, which increases the likelihood of falsely identifying a factor as a predictor. Due to the limitations of biological testing, it is possible that the observed positive chlamydia result was a false positive and/or that one or more of the observed negatives were false negatives. Finally, our behavioural data are based on self-report and participants may have misrepresented their behaviour in order to make their responses more socially desirable[51].

## Conclusions

Despite the limitations described above, our findings can help to improve the uptake of home-based chlamydia testing programs. Home-based chlamydia testing is most attractive for those sexually active young people who report engaging in sexual risk behaviours, so programs targeted to high risk groups will be more successful than programs directed at the general population. Awareness that chlamydia can be asymptomatic and can cause infertility if left untreated may increase the likelihood young people will accept offers of testing. More research is required to determine whether education campaigns based on these messages can increase uptake of home-based chlamydia testing. Unlike clinic-based opportunistic screening which reaches more women than men, home-based testing appears to be equally attractive to men and women and might therefore be a good way to screen otherwise harder to reach young men. Younger adolescents - and those who live with their parents - may find home-testing less attractive, suggesting that alternate screening strategies are required for these groups.

## Competing interests

The authors declare that they have no competing interests.

## Authors' contributions

RSD coordinated the urine testing component of the study, performed the statistical analysis, drafted the manuscript. JG coordinated the behavioural survey component of the study (including recruitment), participated in the design of the study, and helped to draft the manuscript. CK and MH participated in the design of the study and helped to draft the manuscript. All authors read and approved the final manuscript.

## Pre-publication history

The pre-publication history for this paper can be accessed here:

http://www.biomedcentral.com/1471-2458/10/376/prepub
